# Genomic insights into cryptic cycles of microbial hydrocarbon production and degradation in contiguous freshwater and marine microbiomes

**DOI:** 10.1186/s40168-023-01537-7

**Published:** 2023-05-12

**Authors:** Adrien Vigneron, Perrine Cruaud, Connie Lovejoy, Warwick F. Vincent

**Affiliations:** 1 Département de Biologie, Université Laval, Québec, QC Canada; 2 Centre d’études nordiques (CEN), Université Laval, Québec, QC Canada; 3 Institut de Biologie Intégrative et des Systèmes, Université Laval, Québec, QC Canada; 4 Takuvik Joint International Laboratory, CNRS / Université Laval, Québec, QC Canada; 5 Département de Biochimie, de Microbiologie et de Bio-Informatique, Université Laval, Québec, QC Canada; 6 Québec Océan, Université Laval, Québec, QC Canada

**Keywords:** Alkanes, Hydrocarbons, Short hydrocarbon cycle, Biogeochemical cycles, Lakes, Sea

## Abstract

**Background:**

Cyanobacteria and eukaryotic phytoplankton produce long-chain alkanes and generate around 100 times greater quantities of hydrocarbons in the ocean compared to natural seeps and anthropogenic sources. Yet, these compounds do not accumulate in the water column, suggesting rapid biodegradation by co-localized microbial populations. Despite their ecological importance, the identities of microbes involved in this cryptic hydrocarbon cycle are mostly unknown. Here, we identified genes encoding enzymes involved in the hydrocarbon cycle across the salinity gradient of a remote, vertically stratified, seawater-containing High Arctic lake that is isolated from anthropogenic petroleum sources and natural seeps. Metagenomic analysis revealed diverse hydrocarbon cycling genes and populations, with patterns of variation along gradients of light, salinity, oxygen, and sulfur that are relevant to freshwater, oceanic, hadal, and anoxic deep sea ecosystems.

**Results:**

Analyzing genes and metagenome-assembled genomes down the water column of Lake A in the Canadian High Arctic, we detected microbial hydrocarbon production and degradation pathways at all depths, from surface freshwaters to dark, saline, anoxic waters. In addition to Cyanobacteria, members of the phyla Flavobacteria, Nitrospina, Deltaproteobacteria, Planctomycetes, and Verrucomicrobia had pathways for alkane and alkene production, providing additional sources of biogenic hydrocarbons. Known oil-degrading microorganisms were poorly represented in the system, while long-chain hydrocarbon degradation genes were identified in various freshwater and marine lineages such as Actinobacteria, Schleiferiaceae, and Marinimicrobia. Genes involved in sulfur and nitrogen compound transformations were abundant in hydrocarbon producing and degrading lineages, suggesting strong interconnections with nitrogen and sulfur cycles and a potential for widespread distribution in the ocean.

**Conclusions:**

Our detailed metagenomic analyses across water column gradients in a remote petroleum-free lake derived from the Arctic Ocean suggest that the current estimation of bacterial hydrocarbon production in the ocean could be substantially underestimated by neglecting non-phototrophic production and by not taking low oxygen zones into account. Our findings also suggest that biogenic hydrocarbons may sustain a large fraction of freshwater and oceanic microbiomes, with global biogeochemical implications for carbon, sulfur, and nitrogen cycles.

Video Abstract

**Supplementary Information:**

The online version contains supplementary material available at 10.1186/s40168-023-01537-7.

## Background

Hydrocarbons are widespread in the ocean, with natural oil seeps and human activities releasing between 0.47 and 8.3 million tonnes of petroleum annually [1]. These thermogenic compounds, produced over millennia, strongly affect the ecology of marine ecosystems and trigger activity by numerous aerobic and anaerobic oil-degrading microorganisms [2]. Microbial degradation of hydrocarbons in marine environments has been extensively studied in deep-sea sediment seepages [3–7], and following oil spills [8–10]. Detailed investigations following the Deepwater Horizon oil spill in the Gulf of Mexico revealed multiple naturally occurring microbial lineages able to degrade hydrocarbons, and this list is expanding rapidly with metagenomic sequencing and data analysis. For example, mining of the Genome Taxonomy Database (GTDB; gtdb.ecogenomic.org) for hydrocarbon degradation genes revealed that 19% of the 31,900 genomes of this database could potentially degrade hydrocarbons, spanning 24 bacterial phyla [11]. Oxygen conditions, and more broadly the redox potential of the environment, influence the diversity and metabolic pathways of hydrocarbon degraders [2]. In addition, hydrocarbon composition has also been identified as a major structuring factor of the hydrocarbon-degrading community and metabolism, highlighting the importance of substrate preferences and specialization within hydrocarbon-degrading populations [7].

In addition to this long-term cycle of petroleum production and degradation, a cryptic and short-term hydrocarbon cycle has been proposed to widely occur in the ocean [12]. In this short cycle, hydrocarbons in the form of long-chain alkanes such as *n*-pentadecane and *n*-heptadecane are produced by Cyanobacteria such as *Prochlorococcus* and *Synechococcus* species, which are the most abundant cyanobacteria in the ocean [13] and planktonic algae [14, 15] through the activity of aldehyde deformylating oxygenase and fatty acid photodecarboxylase enzymes respectively. Extrapolated to the volume of the ocean and the distribution of *Prochlorococcus* and *Synechococcus*, for which hydrocarbon production rates have been measured, oceanic hydrocarbon production could be up to 500-fold larger than abiotic hydrocarbon releases with up to 300 and 800 million tonnes of alkanes per year [12, 16]. The ecological function or potential benefit of hydrocarbon production for the small pico-sized cyanobacteria remains unclear. Alkanes might increase their membrane fluidity to counter cold or salinity stresses or enhance the efficiency of their light-harvesting thylakoids [17, 18]. As these cyanobacterial hydrocarbons are not found to accumulate in the ocean, alkane loss rates would be equivalent to the production rates, leading to the hypothesis that alkane-producers and degrading microorganisms occupy the same habitat, with potentially rapid cycling of hydrocarbon production and degradation. However, the diversity and metabolic pathways involved in this putative recycling of biogenic long-chain alkanes remain unexplored. Some cultivated oil-degrading aerobic microorganisms such as *Alcanivorax* have been found to degrade pentadecane [16] and to bloom following cyanobacterial pentadecane amendments in seawater incubations [12]. However, there is a gap in knowledge of in situ diversity of bacterial hydrocarbon degraders in the absence of petroleum (thermogenic) hydrocarbons that confound interpretations. Similarly, the contribution of non-phototrophic microorganisms to hydrocarbon production in marine waters has been little explored, yet the aphotic zone represents 90% of the ocean volume, and non-cyanobacterial hydrocarbon production genes, such as the Ole operon, have been identified [19]. In this operon, *oleC* codes for an olefin beta-lactone synthase that catalyzes the head-to-head condensation of fatty acids, leading to the biosynthesis of olefins/alkenes that potentially provides membrane protection toward cold temperatures [20]. In addition, the distribution of this cryptic hydrocarbon cycle along oxygen, salinity, and light gradients of aquatic ecosystems has not been addressed to date, notably under freshwater conditions where Cyanobacteria also flourish and biogenic hydrocarbon production by microalgae has been also observed [21, 22].

**Fig. 1:**
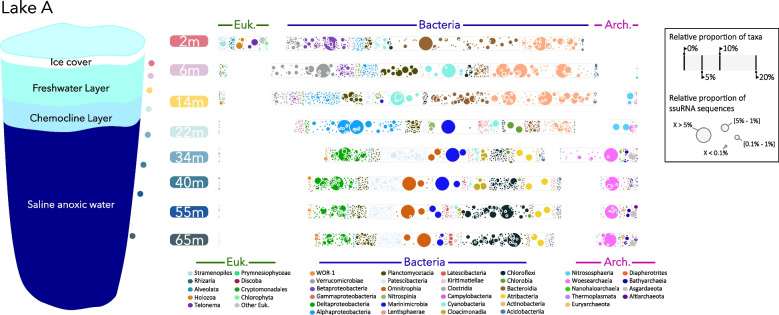
Microbial eukaryotic (Euk) and prokaryotic (Bacteria and Archaea (Arch)) community composition in different layers of permanently stratified Lake A. Each bubble represents a different genus, based on ssuRNA extracted from the metagenomic datasets. The size of the bubbles indicates the relative proportion of each genus in the overall ssuRNA sequences coverage at each depth

The polar regions host numerous marine-derived lakes that formed following the isostatic rebound of the continents triggered by the melting of the ice sheets. Physically isolated from the ocean, freshwater from melting snow and ice accumulates over the marine water trapped in these basins. Due to the strong salinity and temperature differences between these waters, these lakes are highly stratified, with a mixolimnion consisting of the oxic surface freshwater layer immediately beneath the ice and an anoxic (oxygen below detection; < 1 μM) marine monimolimnion at the bottom. The chemocline at the interface between these layers is the zone of highest chemical reactivity, associated with elevated microbial activities [23, 24]. Lake A is one such lake at the extreme north of the Canadian High Arctic, and it provides a remote pristine ecosystem and natural laboratory to investigate microbial community assemblages and metabolism. The microbiome of Lake A has been previously investigated [25–27], revealing a marked stratification of stable and active microorganisms and associated metabolic potentials that align along the salinity, light, and redox gradients of the water column (Fig. 1).

Lake A has a surface layer of freshwater, but > 80% of its 128-m-deep water column is saline, reflecting its origin from the Arctic Ocean [26]. This stratified ecosystem has physical, chemical, and microbiological similarities with freshwater, marine, hadal, and anoxic deep-sea environments, and it therefore offered an outstanding opportunity to investigate the genomic potential for hydrocarbon cycling over a broad range of environmental conditions that are relevant to various marine and freshwater ecosystems and without interference from anthropogenic contaminants or natural seepages. Specifically, we analyzed metagenomes from throughout the water column to address the questions: (i) are biogenic hydrocarbon genes present and how are they distributed across the pronounced vertical gradients of the Lake A environment, (ii) what is the associated microbial diversity, (iii) how would these hydrocarbons be degraded in the system, and (iv) are these hydrocarbon cycles linked to other biogeochemical cycles?

## Results

### Biogenic hydrocarbon producing genes

**Fig. 2:**
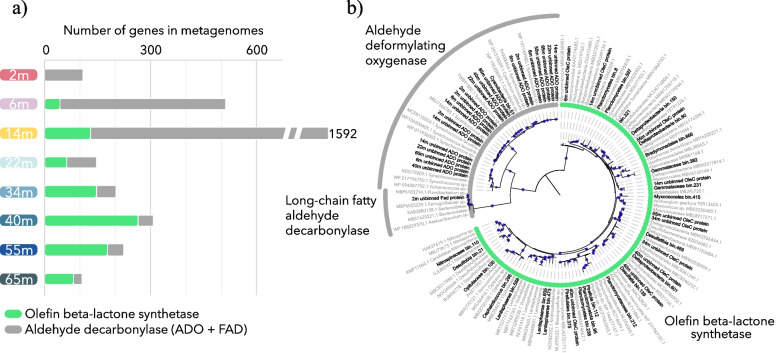
Metabolic potential for hydrocarbon production in Lake A. **a** Number of genes involved in hydrocarbon production in the metagenomes throughout the water column. Aldehyde decarbonylase in gray represents the cumulative alkane production genes (ADO and FAD). **b** Phylogenetic tree of hydrocarbon-producing proteins detected in the metagenomes (in black) including binned and unbinned sequences. Only nonredundant sequences were represented in the tree. Sequences in gray were recovered from NCBI database. Blue dots indicate bootstrap values higher than 0.8

Lake A metagenomes from eight depths (2 to 65 m) were sequenced, normalized, and then analyzed for genes coding for algal fatty acid photodecarboxylase (FAP), squalene synthase (SSL), fatty aldehyde decarbonylase (FAD), aldehyde deformylating oxygenase (ADO), and olefin beta-lactone synthetase (OleC). While FAP, FAD, and ADO enzymes confer the capability to produce long-chain alkanes [28], OleC catalyze the production of olefinic hydrocarbons (alkenes) [19, 29], and SLL is involved in tetraterpenoid hydrocarbon production in the green alga *Botryococcus braunii* [21]. No algal hydrocarbon genes (neither FAP nor SLL) were detected; however up to 3200 prokaryotic genes (unique genes multiplied by their numbers of copies) for hydrocarbon-producing proteins were identified in the dataset. FAD genes phylogenetically related to *Flavobacteriaceae* were identified at in the upper freshwater layer at 2 m (Fig. 2). Numerous ADO genes, affiliated to the phylum *Cyanobacteria*, were detected in the freshwater aerobic layer and the chemocline, with up to 1463 genes at 14 m depth, corresponding to the bottom of the euphotic zone (Fig. 2). By contrast, *oleC* genes were more abundant in the saline anoxic (< 1-μM oxygen) water with a maximum of 263 genes at 40 m. Phylogenetic analysis of *oleC* genes indicated that most of the detected sequences were related to *Deltaproteobacteria* (*Desulfobacteraceae*, *Desulfuromonadales*, and *Myxococcales*) and the PVC superphylum (*Planctomycetes*, *Verrucomicrobia*, *Lentispheraceae*, and *Gemmataceae*). Below 40 m depth, the number of hydrocarbon-producing genes declined with depth, down to 23 ADO and FAD genes and 81 *oleC* genes at 65 m (Fig. 2).

### Hydrocarbon-degrading genes

**Fig. 3:**
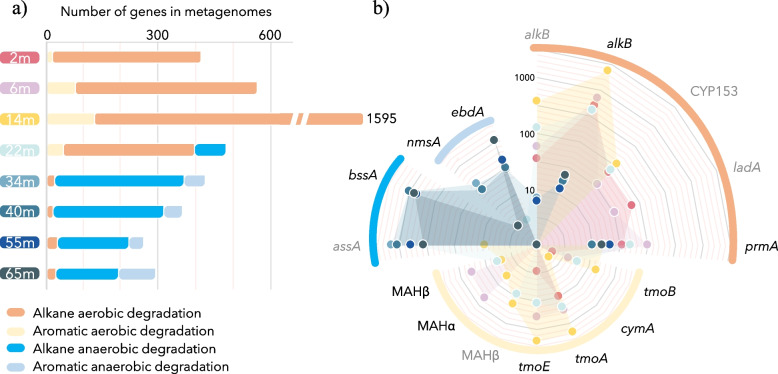
Metabolic potential for hydrocarbon degradation in Lake A. **a** Number of genes involved in hydrocarbon degradation in the metagenomes throughout the water column. Alkane aerobic degradation sums the number of genes of *alkB*, *CYP153*, *ladA*, and *prmA*. Aromatic aerobic degradation sums the number of genes of *tmoABE*, *cymA*, and MAHαβ. Alkane anaerobic degradation is the mean of *assA* and *bssA* gene numbers. Aromatic anaerobic degradation sums the number of genes of *nmsA* and *ebdA*. **b** Relative abundance of each hydrocarbon-degrading genes identified in Lake A. Color of the dots and shades represents the water depth as in **a**. Genes labeled in gray (*assA*, *alkB*, *CYP153*, *ladA*, and MAHβ) were identified using custom HMM profiles as described in Khot et al. [11], whereas genes labeled in black were identified using IMG-MR pipeline. Scale is logarithmic

Overall, 4670 genes coding for proteins involved in hydrocarbon degradation pathways were detected (Fig. 3). The total number of known genes for hydrocarbon-degrading proteins identified in the metagenomes exceeded those of hydrocarbon production at each analyzed depth, except at 14 m where the number of genes of both degrading and producing pathways was maximal and similar (1595 vs 1592). Consistent with the oxygen profile of the water column, aerobic alkane degradation genes (*alkB*, *CYP153*, *ladA*, *prmA*) were abundant from the surface to 22 m, with a maximum of 1451 genes at 14 m (Fig. 3). By contrast, anaerobic alkane degradation genes (*assA*, *bssA*) predominated in the anoxic saline waters with up to 380 genes at 34 m before slowly declining with depth, supporting the absence of deep natural seepages at the bottom of Lake A. The number of aromatic hydrocarbon degradation genes increased with depth until 14 m for aerobic pathways (*tmoABE*, *cymA*, and MAHαβ), and throughout the water column for anaerobic pathways (*ebdA*, *nmsA*), reaching 109 genes at 65 m (Fig. 3).

### Hydrocarbon cycling microbial populations

**Fig. 4:**
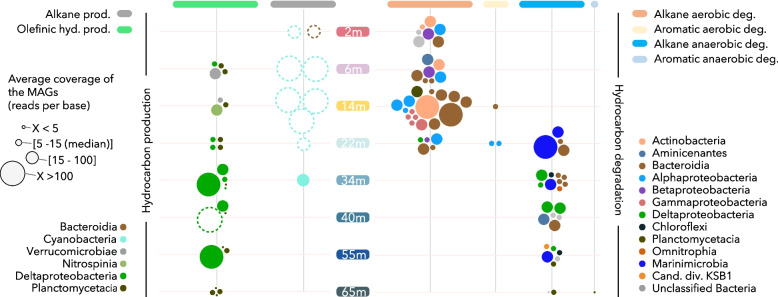
Depth distribution of metagenome-assembled genomes (MAGs) with hydrocarbon production (prod.) and degradation (deg.) potential recovered from the Lake A metagenomic dataset. Alkane prod., ADO and FAD genes; olefinic hyd. prod., *oleC* gene; alkane aerobic deg., *alkB*, *CYP153*, *ladA*, and *prmA* genes; aromatic aerobic deg., *tmoABE*, *cymA*, and MAHαβ genes; alkane anaerobic deg., *assA* and *bssA* genes; aromatic anaerobic deg., *nmsA* and *ebdA* genes. The size of the dots indicates the relative abundance of each MAG, and dots are color coded by their taxonomy. Dashed clear dots represent contigs with hydrocarbon-producing genes that were not binned into MAGs

A total of 250 MAGs with > 40% completeness and < 5% contamination levels were recovered from the combined metagenomic dataset. Among them, 89 MAGs (35.6%) harbored genes for hydrocarbon cycling (Fig. 4 and Supplementary Fig. S1). ADO genes were identified in only one MAG affiliated with marine *Cyanobacteria* recovered from the lower chemocline zone. Nonetheless, ADO genes with high coverage were also identified at 6- and 14-m samples in unbinned contigs taxonomically affiliated with the cyanobacterial taxon *Synechococcus*. In addition, one contig from the 2-m metagenomic dataset and related to *Flavobacteriaceae* (*Bacteroidia*) included a long-chain fatty aldehyde decarbonylase gene (Fig. 4). Olefinic hydrocarbon production genes were identified in 26 MAGs, of which 14 were assigned to PVC lineages (Planctomycetota, *Pirellula*, *Lentisphaerae*, *Gemmataceae*, and *Opitutaceae*) and 8 were related to *Deltaproteobacteria* (Fig. 4 and Supplementary Fig. S1). Olefinic hydrocarbon producers in the oxic freshwater were related to PVC and *Nitrospinaceae*, whereas highly abundant *Deltaproteobacteria* MAGs were predominant in anoxic saline waters. Six low abundance PVC MAGs with *oleC* were also detected at the deepest depth (Supplementary Fig. 1).

Hydrocarbon degradation pathways were identified in 63 taxonomically diverse MAGs (Fig. 4 and Supplementary Fig. S1). Aerobic alkane degradation genes (*alkB*, *CYP153*) were detected in *Actinobacteria* (*Nanopelagicales*), *Bacteroidetes* (*Sediminibacterium*, *Schleiferiaceae*) and *Alpha*-(*Rhodobacteraceae*), *Beta*- (*Polaromonas*), and *Gamma*- *proteobacteria* (SAR86, *Porticoccaceae*, *Woeseiaceae*, *Pseudohongiellaceae*). Long-chain alkane monooxygenase genes (*ladA*) were also identified in *Actinobacteria* (*Microbacteriaceae*, *Alpinimonas*) recovered at 2 m. Aerobic aromatic hydrocarbon degradation pathways were detected in *Alphaproteobacteria* (*Rhodospirillales*) and in *Rhodothermales* MAGs. Based on the average coverage of the MAGs, *Nanopelagicales* and *Schleiferiaceae* were the most abundant aerobic hydrocarbon-degrading lineages of the system (Fig. 4 and Supplementary Fig. S1). In addition, anaerobic alkane degradation pathway (*assA* and *bssA*) genes were identified in *Marinimicrobia,* *Deltaproteobacteria* (*Desulfobacteraceae*, *Desulfatiglans*, *Syntrophales*), *Bacteroidetes*, *Chloroflexi* as well as in poorly characterized lineages (candidate division KSB1, *Aminicenantes*), and *Abyssubacteria* that also possessed aromatic hydrocarbon degradation genes. Coverage of the MAGs indicated that populations of Marinimicrobia were the most abundant anaerobic hydrocarbon degraders in low oxygen waters (Fig. 4 and Supplementary Fig. S1).

### Sulfur and nitrogen cycle genes in hydrocarbon short cycle populations

Genome analysis of the hydrocarbon short cycle populations indicated that 86.5% of the MAGs harbored inorganic nitrogen cycling (70% of the MAGs) or sulfur cycling (71% of the MAGs) genes, and 54% included genes from both cycles (Supplementary Fig. S1). Nitrite and dissimilatory nitrate reduction pathways were the most represented pathways in hydrocarbon producers, notably in *Planctomycetes*, *Nitrospina*, and *Deltaproteobacteria* (Myxococcota, *Desulfatibia*, and *Desulfobia*). The *Cyanobacteria* MAG also included the potential for nitrate and urea assimilation. By contrast, genes coding for enzymes involved in the oxidation of sulfide (SQR) and thiosulfate (*doxD*, *tsdA*) and sulfate reduction (*aprAB*, *dsrAB*) were the most detected in hydrocarbon degraders. Potential sulfide oxidizers included members of the *Marinimicrobia*, *Bacteroidales*, and *Alphaproteobacteria* lineages, whereas thiosulfate oxidizers were related to other *Bacteroidetes* lineages (*Flavobacteriales* including *Scheiferiaceae*, *Cytophagales*, and *Cryomorphaceae*). Sulfate reduction pathway genes were identified in *Desulfatiglandales*, *Syntrophales*, *Desulfobacteraceae*, *Chloroflexi*, and *Abyssubacteria*. In addition, dissimilatory nitrate reductase and sulfur oxidation genes were also identified in the highly dominant *Nanopelagicales* MAG with hydrocarbon degradation genes, whereas in the highly dominant *Marinimicrobia* MAGs, sulfur oxidation genes were identified along nitrous-oxide reductase genes (Supplementary Fig. S1).

## Discussion

### Freshwater hydrocarbon short cycle

The upper water layer (0–12 m) of meromictic Lake A is derived from snow and ice melt, resulting in a perennial freshwater environment (salinity < 0.7 ppt) [30]. This was consistent with the microbial community composition being dominated by freshwater taxa, including *Flavobacteriales* (*Bacteroidia*), *Burkholderiales* (*Betaproteobacteria*), *Nanopelagicales* (ex *Actinobacteria* acl), and *Cyanobacteria* (Fig. 1), which are frequent major lineages in cold lakes and rivers [31, 32]. These lineages were previously detected using 16S rRNA sequencing, indicating a ribosomal activity at these depths [25]. *Cyanobacteria* have been also detected in the anoxic dark waters of Lake A, as in other polar meromictic lakes [33], and picocyanobacteria are a major constituent of the phototrophic plankton [34]. Eukaryotic algae were also identified, supporting previous 18S rRNA gene sequencing [35] but represented a minor fraction of the total community reads. No algal gene encoding hydrocarbon production was detected, suggesting a minor contribution to hydrocarbon biogenic production. Known hydrocarbon-producing algae have been isolated from temperate to tropical freshwater environments [21], suggesting that these lineages might not be adapted to the cold temperatures. Multiple genes of long-chain fatty aldehyde decarbonylase (FAD) and aldehyde deformylating oxygenase (ADO), both producing C_16_–C_18_ alkanes [11], were identified in the 2-m and 6-m metagenomes, suggesting a strong potential for bacterial long-chain alkane production in the upper freshwater layer of the lake. Based on the taxonomic affiliation of the contigs and MAGs with those genes, lacustrine *Flavobacteriaceae* and *Cyanobacteria* populations were likely the main hydrocarbon producers in this freshwater habitat. This indicates a new ecological role for *Flavobacteriaceae* members in these ecosystems and suggests that the potential for hydrocarbon production in *Cyanobacteria* is not limited to marine species, providing field confirmation of results based on *Cyanobacteria* culture studies [36].

**Fig. 5:**
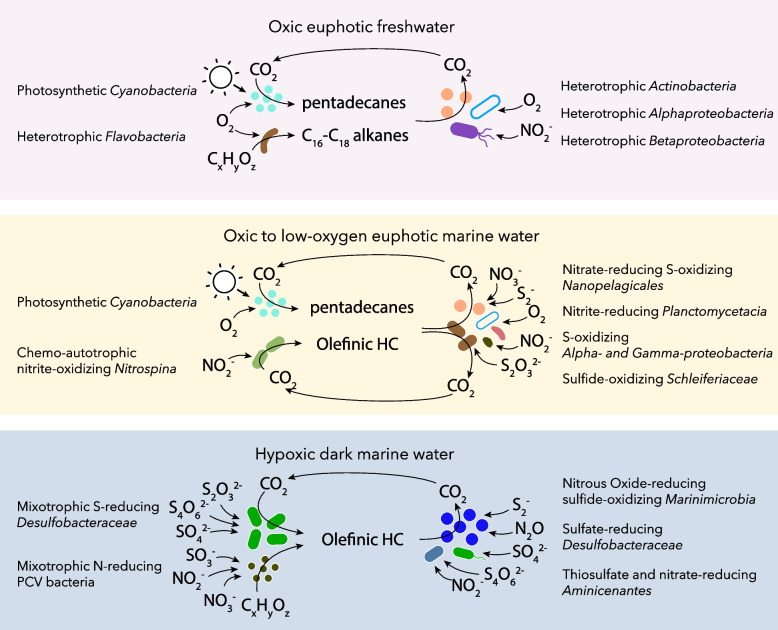
Conceptual model of hydrocarbon short cycles identified along oxygen, salinity, and light gradients of the Lake A water column. Schematic bacteria are colored as in Fig. 4 color code

Consistent with the bacterial production of long-chain alkanes, genes coding for C_15_–C_36_ alkane monooxygenase (*ladA*), conferring the ability to degrade long-chain alkanes, were also detected at 2 m and 6 m. The identification of these genes in two MAGs affiliated to *Actinomycetales* and *Alpinimonas* (*Microbacteriaceae*) that were recovered at 2 m suggests that long-chain alkanes produced by the *Flavobacteriaceae* and *Cyanobacteria* could be readily degraded by members of the *Actinobacteria* phylum (Fig. 5). Likewise, numerous genes coding for alkane monooxygenase (AlkB), and in lesser proportion cytochrome P450 alkane hydroxylase (CYP153), were also detected in the freshwater layer. Identified in MAGs affiliated to *Burkholderiales*, *Sediminibacterium*, *Alphaproteobacteria*, and *Nanopelagicales*, these genes code for enzymes that confer the ability to degrade a large variety of (C_5_–C_16_) alkanes and alkenes that fall within the range of fatty aldehyde decarbonylase and aldehyde deformylating oxygenase products. By contrast, genes encoding for shorter hydrocarbon (methane, butane, propane) oxidation and aromatic hydrocarbon degradation were either not or little detected in the water column, suggesting that the hydrocarbon catabolism is centered on bacterial long-chain alkanes. Together, these results indicate that long-chain hydrocarbon cycling, suspected to occur in oceans, also occurs in the low-conductivity surface layer of Lake A, extending the potential niche for this cycle to freshwaters. Fuelled by predominant and widespread freshwater lineages of *Cyanobacteria* and *Flavobacteriales*, then recycled by *Nanopelagicales* and *Burkholderiales* that also represent major components of the freshwater microbiome [32, 37], this freshwater hydrocarbon short cycle is likely to occur in other cold lakes and rivers. Our results suggest the need to extend the range of ecological functions carried out by the dominant lineages of freshwater bacteria, with a new potential role in the previously unconsidered hydrocarbon short cycle that could represent a major carbon source, notably in oligotrophic waters.

### Hydrocarbon biosynthesis under aphotic and low oxygen conditions

The depth distribution of ADO genes, as well as the high coverage of ADO gene-bearing contigs and MAGs related to *Synechococcus*, suggests that *Cyanobacteria* are the major hydrocarbon producers in the euphotic, upper saline waters of Lake A. This result is consistent with previous studies of cultivated isolates from the ocean that estimated a production of up to 649 Tg of pentadecane per year by *Cyanobacteria* [12]. In addition, we also detected the olefinic/alkene hydrocarbon synthesis pathway in the euphotic and oxic marine water in a *Nitrospina* MAG with nitrite oxidation pathways (*nirADK*, *nxrAB*). *Nitrospina* species are widespread nitrifying bacteria in the ocean and play a major role in the marine nitrogen cycle by oxidizing nitrite [38]. This observation suggests that non-photosynthetic but ecologically important microorganisms could also contribute to the biogenic long-chain alkene pool in the ocean (Fig. 5). Finally, our detection of numerous *oleC* genes in the deep anoxic seawater of Lake A suggests that bacterial production of hydrocarbons is not limited to the euphotic zone but could also occur under aphotic and low oxygen conditions. Phylogenetic analysis of *oleC* genes (Fig. 2) and mining of the Lake A MAGs (Fig. 4) revealed that various *Deltaproteobacteria* and *Planctomycetes* populations may contribute to olefinic hydrocarbon production in this environment. Members of *Candidatus* Desulfaltia and Desulfatibia, which were identified as the most abundant populations with olefinic hydrocarbon production genes in the ecosystem, have been previously detected in significant proportions in low oxygen marine waters (< 1 μM) [39], suggesting that olefinic hydrocarbon production could represent an important source of hydrocarbons in oceanic oxygen minimum zones. These hypoxic/anoxic zones represent 10 million km^3^ or approximately 1% of the ocean volume and are expanding in oceans [40]. Our results indicate that olefinic hydrocarbon production from non-phototrophic microorganisms in oceans could increase in the future. Together, our findings suggest that the current estimation of bacterial hydrocarbon production could be substantially underestimated by neglecting non-cyanobacterial production and by not taking low oxygen zones into account.

### Biohydrocarbon degradation in petroleum-free marine ecosystems

Due to its remote location and permanent ice cover, pristine Lake A and its trapped seawater are free of natural seepage and chemical pollution, providing a unique opportunity to elucidate the identity of microorganisms involved in the marine hydrocarbon short cycle without interference from specialist petroleum and oil-degrading microorganisms. The chemocline of the lake had numerous chemical (oxygen, light, salinity) and microbial (e.g., dominance of lineages related to *Pelagibacter*, *Synechococcus*, *Marinimicrobia*, SAR86, *Flavobacteria* NS3, *Thaumarchaeota*) similarities with marine systems (Fig. 1), allowing extrapolation to similar oceanic biomes. Based on the detection of alkane monooxygenase gene (*alkB*) in MAGs and the average coverage of the contigs, our results indicate that marine populations of Schleiferiaceae and *Actinomycetales* would be the predominant hydrocarbon degraders in saline-oxygenated waters, followed by a population of marine Planctomycetota and various alpha (*Rhodobacteraceae*, *Parasphingorhabdus*, SHVP01) and gamma-proteobacterial populations (*Woeseiaceae*, ex JTB255 and SAR86), frequently and ubiquitously identified in marine environments [41–43]. Although these genomic results need to be confirmed by activity-based approaches, multiple marine *Actinobacteria* strains and cultivated marine species of the family Schleiferiaceae degrade hydrocarbons under aerobic conditions [44, 45]. However, our results strongly contrast with previous experiments that proposed bloom-forming, oil-degrading microorganisms *Alcanivorax* and *Thalassolituus* as potential biogenic hydrocarbon degraders [12]. Previous studies that identified hydrocarbon degraders using enrichment of the Gulf of Mexico or North Atlantic subtropical gyre waters with pentadecane might have been biased by petroleum hydrocarbon contamination or by the priming effect of these contaminants, and similar experiments with Lake A inoculate would provide a better background survey of naturally occurring pentadecane degraders.

Our analysis of the trapped anoxic marine waters of Lake A revealed that populations of *Marinimicrobia* (previously SAR406) were the most abundant anaerobic hydrocarbon degraders under these conditions, extending the putative metabolic capability of the members of this phylum. Members of this metabolically versatile phylum are widespread in the ocean, flourishing in low oxygen waters and oxygen minimum zones [46]. In the context of global warming and extension of hypoxic zones in oceans, these results suggest a major role of *Marinimicrobia* in the oceanic hydrocarbon short cycle. By contrast, known anaerobic hydrocarbon degraders related to *Desulfatiglandales* [47] represented a minor proportion of the community, suggesting that oil-degrading species are less adapted to the degradation of biogenic long-chain alkanes and confirming the substrate preferences and specialization within the hydrocarbon degrader guild [7]. Together, our data indicate that under both oxic and anoxic conditions, biogenic hydrocarbon degradation mostly implicates different microbial taxa than petroleum hydrocarbon degradation, with a previously unrecognized diversity of microorganisms. The presence of microorganisms with hydrocarbon-degrading potential, such as *Alcanivorax*, in apparently oil-free systems has been previously explained by their metabolic flexibility, allowing them to persist in natural environments by the use alternative substrates such as natural polyesters [48]. Therefore, this study would be usefully extended in the future by geochemical characterization of hydrocarbons in diverse aquatic environments, including Lake A, and by the application of transcriptomics and experimental protocols to assess hydrocarbon cycling activities and rates.

### Hydrocarbon short cycles connect to sulfur and nitrogen cycles

Genome analysis of hydrocarbon short cycle populations revealed a strong interconnection with nitrogen and sulfur cycles (Fig. 5, Supplementary Fig. S1). The most abundant hydrocarbon-degrading lineages *Nanopelagicales* and *Marinimicrobia* were potentially involved in sulfide oxidation coupled to nitrate or nitrous-oxide reduction respectively. Genes coding for the transformation of sulfur cycle intermediates (sulfide, thiosulfate) were also frequently detected in hydrocarbon producer and degraders, including in potentially major hydrocarbon degraders related to *Marinimicrobia* and *Schleiferiaceae* lineages. The results suggest that these substrates fuel a large part of the hydrocarbon short cycle, further underscoring their ecological importance in aquatic ecosystem functioning [25]. Neither nitrate nor nitrite accumulates in Lake A [30]. However, genomic data indicated that nitrite might also play a major role in the hydrocarbon cycle, fuelling the energetic metabolism of various major hydrocarbon producers (*Planctomycetes*, *Myxococcota*, and *Nitrospina*) and degraders (*Sedimentisphaerales*, *Vicinamibacterales*, *Betaproteobacteria*). These results suggest a rapid depletion by microbial communities and add another line of evidence for the central role of nitrite in ocean geochemistry [50, 51]. They also indicate that biogeochemical intermediates in oxidative and reductive processes may occupy a major place in the hydrocarbon short cycle. Although energetically less efficient than end products, utilization of sulfur and nitrogen cycle intermediates may allow the development of microorganisms over a broader spectrum of environmental gradients, supporting a wide distribution of the hydrocarbon short cycle in aquatic environments, as suggested by our observations.

## Conclusions

Our metagenomic analysis of hydrocarbon cycling genes and populations focused on a petroleum-free model ecosystem that is physically, chemically, and microbiologically relevant to many aquatic biomes. The results show that the hydrocarbon short cycle is not limited to marine waters but likely occurs under a broad range of salinity and oxygen concentrations, from oxic freshwaters to anoxic marine conditions (Fig. 5). Entangled with sulfur and nitrogen cycles, the hydrocarbon short cycle involved diverse microorganisms, including lineages that are common and often predominate in freshwater and marine ecosystems. Although *Cyanobacteria* were found as major potential drivers of the hydrocarbon production in the euphotic zone, our results also revealed that widely distributed lineages of the *Flavobacteriaceae*, PVC, *Nitrospina*, and *Deltaproteobacteria* phyla could contribute to biogenic hydrocarbon production (Fig. 5). Analysis of hydrocarbon degradation genes highlighted that the degradation of bacterial alkanes involved a large diversity of microbial lineages that differ from petroleum hydrocarbon-degrading species. Since the efficiency and rapidity of the microbial response to oil inputs could be linked to the history of contamination [52], a major consequence of this decoupling is that hydrocarbons produced by bacteria might have little or no priming effect on attenuating oil spills. The ability of these microorganisms to degrade petroleum hydrocarbons should be investigated experimentally to test this hypothesis. However, particular attention must be paid to pristine environments, such as High Arctic ecosystems that are increasingly threatened by oil exploitation and shipping, to prevent any oil contamination that might remain undegraded by the native microbial population.

## Methods

### Site description, sample collection, and nucleic acid extraction

Lake A is located at the far northern coast of Ellesmere Island, Nunavut, in the Canadian High Arctic (latitude 82° 59.667′ N, longitude 75° 26.602′ W). It is a perennially ice-covered, highly stratified meromictic lake, with its bottom saline layer derived from Arctic Ocean seawater that was trapped by isostatic uplift of the land around 3000 years ago [30]. The water column has an oxygenated freshwater layer (14.1 mg.L^−1^ of oxygen; salinity < 0.7 ppt) that extends from under the ice down to 12 m depth, a chemo- and halocline located below the freshwater down to 24 m, and anoxic marine-derived saline waters (salinity > 30 ppt; oxygen below detection limit of the profiler) below 24 m, containing high concentrations of sulfate (~ 20 mM) and sulfide (~ 0.3 mM). Light penetrates through the ice to the water column beneath, with an oxygen-containing euphotic zone that extends to 22 m.

Water samples were collected in summer 2017 from three separate 24-cm-diameter holes drilled in the center of Lake A through its 0.6-m-thick ice cover. The sampling strategy and experimental procedures used in this study were previously detailed [25]. Briefly, 1 l of water was collected at eight different depths (2 m, 6 m, 14 m, 22 m, 34 m, 40 m, 55 m, and 65 m) in the three holes, spanning the oxygen, salinity, and temperature gradients of the water column. Triplicate water samples were filtered through separate 0.22-µm pore size Sterivex filters™ (Merck Millipore) and then stored below − 50 °C until nucleic acid extraction. Nucleic acids were extracted using the Qiagen Allprep DNA/RNA Mini Kit with modifications [53].

### Metagenomic library preparation, sequencing, and analysis

One metagenomic library per sampled depth was prepared using Illumina Nextera XT kit, and each library was sequenced in two Illumina MiSeq (2 × 300 bp) runs and one Illumina NexSeq run (2 × 150 bp) at the Institut de Biologie Integrative et des Systèmes (IBIS) sequencing platform (Université Laval, Canada) and at the CGEB — Integrated Microbiome Resource (Dalhousie University, Canada) respectively. Datasets were quality filtered using Trimmomatic v.0.39 [54]. Reads of ribosomal small subunit were extracted from metagenomic reads using Infernal v.1.1.4 [55], and taxonomic affiliation of the extracted 16S rRNA reads longer than 100 bp was determined using BLAST against Silva database release 138 as reference [56]. Since metagenomic 16S rRNA reads spanned various regions of the 16S rRNA gene, taxonomic assignments were limited to the genus level. For each sample, quality-filtered metagenomic reads from the different sequencing runs were pooled and assembled using SPAdes (option meta) [57]. Assembled contigs longer than 200 bp and mapping files (BAM files generated using BBmap [58]) were uploaded to the IMG/MR platform for gene calling and functional annotation using pipeline v.4.16.6 [59]. Genes involved in hydrocarbons degradation were also identified using HMM profiles of the CANT-HYD database with noise cut-off thresholds [11]. For additional information, the detailed description of hydrocarbon-degrading genes and pathways in the CANT-HYD database could be consulted [11]. To account for differences in sequencing depth between samples, metagenomes were normalized to the size of the smallest dataset (2 m: 559,162 genes).

For metagenome-assembled genome reconstruction, all quality-filtered sequences were pooled and co-assembled using MEGAHIT v.1.2.9 [60]. Read coverage of the contigs was carried out using bwa-mem (http://bio-bwa.sourceforge.net), followed by contig binning using MetaBAT-2 [61] with contigs longer than 2000 bp. The completeness and contamination level of the MAGs were evaluated using CheckM [62]. Only bins with a contamination level under 5% and completeness above 40% were analyzed (Supplementary Fig. S1). Taxonomic affiliation of the MAGs was carried out using GTDB-tk [63]. Hydrocarbon cycling genes in the MAGs were identified using IMG/MR annotations and CANT-HYD HMM profiles as for metagenomic dataset. Relative abundance of the MAGs was estimated with the average coverage of the contigs determined using bwa-mem and averaged using the jgi_summarize_bam_contig_depths script. Genetic composition of the MAGs was determined using KEGG Orthologies (KO). A list of KOs detected per MAG is available in Supplementary dataset 1.

For phylogenetic analysis of hydrocarbon-producing genes, amino acid sequences of fatty aldehyde decarbonylase, aldehyde deformylating oxygenase, and olefin beta-lactone synthetase were recovered from the metagenomic dataset and compared against NCBI database. Sequences with the best hit of blast were downloaded and aligned with the metagenomic sequences using Clustal Omega [64]. Maximum likelihood tree of 145 sequences with 725 amino acid sites was constructed using IQ-TREE with 1000 bootstraps and the LG + F + I + G4 model [65].

## Supplementary Information

**Additional file 1: Supplementary Fig. S1.** Detail of the genomic MAGs analysed in this study. Completeness and contamination were determined using ChekM. Maximum coverage was calculated using the average coverage determined using BWA-MEM of all contigs found in the MAGs. Green dots indicate the presence in the MAGs of hydrocarbon production genes while orange and blue dots refer to hydrocarbon degradation genes. Pink and purple dots indicate genes involved in nitrogen and sulfur transformations respectively. Detail of the genes (KO) identified in the MAGs is available in Supplementary Dataset 1.

**Additional file 2: Supplementary Dataset 1.** Detail of the genes (KO) identified in the MAGs involved in hydrocarbon production and degradation.

## Data Availability

Assembled metagenome data are available in IMG/MR (https://img.jgi.doe.gov/mer/) under the following accession numbers: 3300033443, 3300033444, 3300033445, 3300033439, 3300033411, 3300033473, 3300033474, and 3300033495. Co-assembly is also available on IMG/MR under accession number 3300033064. Raw amplicon sequences and bin files were deposited in the NCBI public database under BioProject PRJNA616293 (https://www.ncbi.nlm.nih.gov/bioproject/PRJNA616293). A list of KOs detected per MAG is available in Supplementary dataset 1. Environmental metadata were previously published [25, 49], and additional data are available in the Nordicana D database (http://www.cen.ulaval.ca/nordicanad).

## Abbreviations

ADO: Aldehyde deformylating oxygenase
FAD: Fatty aldehyde decarbonylase
FAP: Fatty acid photodecarboxylase
KO: KEGG Orthology
MAG: Metagenome-assembled genome
NCBI: National Center of Biotechnology Information
PVC: Planctomycetes, Verrucomicrobia, and Chlamydiae

## References

[1] National Research Council. Oil in the sea III: inputs, fates, and effects. The National Academies Press; 2003. Available from: https://www.nap.edu/catalog/10388/oil-in-the-sea-iii-inputs-fates-and-effects 25057607

[2] Head IM, Jones DM, Röling WFM (2006). Marine microorganisms make a meal of oil. Nat Rev Microbiol..

[3] Dong X, Rattray JE, Campbell DC, Webb J, Chakraborty A, Adebayo O (2020). Thermogenic hydrocarbon biodegradation by diverse depth-stratified microbial populations at a Scotian Basin cold seep. Nat Commun.

[4] Bacosa HP, Erdner DL, Rosenheim BE, Shetty P, Seitz KW, Baker BJ (2018). Hydrocarbon degradation and response of seafloor sediment bacterial community in the northern Gulf of Mexico to light Louisiana sweet crude oil. ISME J.

[5] Callaghan AV, Davidova IA, Savage-Ashlock K, Parisi VA, Gieg LM, Suflita JM (2010). Diversity of benzyl- and alkylsuccinate synthase genes in hydrocarbon-impacted environments and enrichment cultures. Environ Sci Technol.

[6] Dombrowski N, Seitz KW, Teske AP, Baker BJ (2017). Genomic insights into potential interdependencies in microbial hydrocarbon and nutrient cycling in hydrothermal sediments. Microbiome.

[7] Vigneron A, Alsop EB, Cruaud P, Philibert G, King B, Baksmaty L (2017). Comparative metagenomics of hydrocarbon and methane seeps of the Gulf of Mexico. Sci Rep.

[8] Mason OU, Hazen TC, Borglin S, Chain PSG, Dubinsky EA, Fortney JL (2012). Metagenome, metatranscriptome and single-cell sequencing reveal microbial response to Deepwater Horizon oil spill. ISME J.

[9] Valentine DL, Fisher GB, Bagby SC, Nelson RK, Reddy CM, Sylva SP (2014). Fallout plume of submerged oil from Deepwater Horizon. Proc Natl Acad Sci.

[10] Dubinsky EA, Conrad ME, Chakraborty R, Bill M, Borglin SE, Hollibaugh JT (2013). Succession of hydrocarbon-degrading bacteria in the aftermath of the Deepwater Horizon oil spill in the Gulf of Mexico. Environ Sci Technol.

[11] Khot V, Zorz J, Gittins DA, Chakraborty A, Bell E, Bautista MA (2022). CANT-HYD: a curated database of phylogeny-derived hidden Markov models for annotation of marker genes involved in hydrocarbon degradation. Front Microbiol.

[12] Love CR, Arrington EC, Gosselin KM, Reddy CM, Van Mooy BAS, Nelson RK (2021). Microbial production and consumption of hydrocarbons in the global ocean. Nat Microbiol.

[13] Huang S, Wilhelm SW, Harvey HR, Taylor K, Jiao N, Chen F (2012). Novel lineages of *Prochlorococcus* and *Synechococcus* in the global oceans. ISME J.

[14] Sorigué D, Légeret B, Cuiné S, Morales P, Mirabella B, Guédeney G (2016). Microalgae synthesize hydrocarbons from long-chain fatty acids via a light-dependent pathway. Plant Physiol.

[15] Sorigué D, Légeret B, Cuiné S, Blangy S, Moulin S, Billon E (2017). An algal photoenzyme converts fatty acids to hydrocarbons. Science.

[16] Lea-Smith DJ, Biller SJ, Davey MP, Cotton CAR, Perez Sepulveda BM, Turchyn AV, et al. Contribution of cyanobacterial alkane production to the ocean hydrocarbon cycle. Proc Natl Acad Sci. 2015;112:13591–6. 10.1073/pnas.1507274112 PMC4640736 26438854

[17] Lea-Smith DJ, Ortiz-Suarez ML, Lenn T, Nürnberg DJ, Baers LL, Davey MP (2016). Hydrocarbons are essential for optimal cell size, division, and growth of Cyanobacteria. Plant Physiol.

[18] Moulin SLY, Beyly-Adriano A, Cuini S, Blangy S, LS, Ly B, Floriani M, et al. Fatty acid photodecarboxylase is an ancient photoenzyme that forms hydrocarbons in the thylakoids of algae. Plant Physiol. 2021;186:1455–72. 10.1093/plphys/kiab168 PMC8260138 33856460

[19] Sukovich D, Seffernick J, Richman J, Gralnick J, Wackett L (2010). Widespread head-to-head hydrocarbon biosynthesis in bacteria and role of Olea. Appl Environ Microbiol.

[20] Sukovich D, Seffernick J, Richman J, Hunt K, Gralnick J, Wackett L (2010). Structure, function, and insights into the biosynthesis of a head-to-head hydrocarbon in *Shewanella oneidensis* Strain MR-1. Appl Environ Microbiol.

[21] Hirano K, Hara T, Ardianor, Nugroho RA, Segah H, Takayama N, et al. Detection of the oil-producing microalga *Botryococcus braunii* in natural freshwater environments by targeting the hydrocarbon biosynthesis gene SSL-3. Sci Rep. 2019;9:16974. 10.1038/s41598-019-53619-y PMC6861321 31740707

[22] Knights BA, Brown AC, Conway E, Middleditch BS (1970). Hydrocarbons from the green form of the freshwater alga *Botryococcus braunii*. Phytochemistry.

[23] Lauro FM, DeMaere MZ, Yau S, Brown MV, Ng C, Wilkins D (2011). An integrative study of a meromictic lake ecosystem in Antarctica. ISME J.

[24] Peduzzi S, Tonolla M, Hahn D (2003). Isolation and characterization of aggregate-forming sulfate-reducing and purple sulfur bacteria from the chemocline of meromictic Lake Cadagno. Switzerland FEMS Microbiol Ecol.

[25] Vigneron A, Cruaud P, Culley AI, Couture R-M, Lovejoy C, Vincent WF (2021). Genomic evidence for sulfur intermediates as new biogeochemical hubs in a model aquatic microbial ecosystem. Microbiome.

[26] Vigneron A, Cruaud P, Lovejoy C, Vincent WF (2022). Genomic evidence of functional diversity in DPANN archaea, from oxic species to anoxic vampiristic consortia. ISME Commun.

[27] Comeau AM, Harding T, Galand PE, Vincent WF, Lovejoy C (2012). Vertical distribution of microbial communities in a perennially stratified Arctic Lake with saline, anoxic bottom waters. Sci Rep.

[28] Choi YJ, Lee SY (2013). Microbial production of short-chain alkanes. Nature.

[29] Wackett LP, Wilmot CM. Chapter 2 - Hydrocarbon biosynthesis in microorganisms. Direct Microb Convers Biomass Adv Biofuels. 2015. p. 13–31.

[30] Gibson JAE, Vincent WF, Van Hove P, Belzile C, Wang X, Muir D (2002). Geochemistry of ice-covered, meromictic Lake A in the Canadian High Arctic. Aquat Geochem.

[31] Cruaud P, Vigneron A, Fradette M-S, Dorea CC, Culley AI, Rodriguez MJ (2019). Annual bacterial community cycle in a seasonally ice-covered river reflects environmental and climatic conditions. Limnol Oceanogr.

[32] Newton RJ, Jones SE, Eiler A, McMahon KD, Bertilsson S (2011). A guide to the natural history of freshwater lake bacteria. Microbiol Mol Biol Rev.

[33] Panwar P, Allen MA, Williams TJ, Hancock AM, Brazendale S, Bevington J (2020). Influence of the polar light cycle on seasonal dynamics of an Antarctic lake microbial community. Microbiome.

[34] Veillette J, Martineau M-J, Antoniades D, Sarrazin D, Vincent WF (2010). Effects of loss of perennial lake ice on mixing and phytoplankton dynamics: insights from High Arctic Canada. Ann Glaciol.

[35] Charvet S, Vincent WF, Comeau A, Lovejoy C (2012). Pyrosequencing analysis of the protist communities in a high arctic meromictic lake: DNA preservation and change. Front Microbiol.

[36] Shakeel T, Fatma Z, Fatma T, Yazdani SS (2015). Heterogeneity of alkane chain length in freshwater and marine cyanobacteria. Front Bioeng Biotechnol..

[37] Neuenschwander SM, Ghai R, Pernthaler J, Salcher MM (2017). Microdiversification in genome-streamlined ubiquitous freshwater Actinobacteria. ISME J.

[38] Luecker S, Nowka B, Rattei T, Spieck E, Daims H (2013). The genome of *Nitrospina gracilis* illuminates the metabolism and evolution of the major marine nitrite oxidizer. Front Microbiol..

[39] van Vliet DM, von Meijenfeldt FAB, Dutilh BE, Villanueva L, Sinninghe Damsté JS, Stams AJM, et al. The bacterial sulfur cycle in expanding dysoxic and euxinic marine waters. Environ Microbiol. 23:2834–2857. 10.1111/1462-2920.15265 PMC8359478 33000514

[40] Paulmier A, Ruiz-Pino D (2009). Oxygen minimum zones (OMZs) in the modern ocean. Prog Oceanogr.

[41] Hoarfrost A, Nayfach S, Ladau J, Yooseph S, Arnosti C, Dupont CL (2020). Global ecotypes in the ubiquitous marine clade SAR86. ISME J.

[42] Mußmann M, Pjevac P, Krüger K, Dyksma S (2017). Genomic repertoire of the *Woeseiaceae*/JTB255, cosmopolitan and abundant core members of microbial communities in marine sediments. ISME J.

[43] Sunagawa S, Coelho LP, Chaffron S, Kultima JR, Labadie K, Salazar G (2015). Structure and function of the global ocean microbiome. Science.

[44] Liu R, Lai Q, Gu L, Yan P, Shao Z. *Croceimicrobium hydrocarbonivorans* gen. nov. sp. nov., a novel marine bacterium isolated from a bacterial consortium that degrades polyethylene terephthalate. Int J Syst Evol Microbiol. 2021;71:004770. 10.1099/ijsem.0.004770 33847553

[45] Rathore DS, Sheikh M, Singh SP (2021). Marine Actinobacteria: new horizons in bioremediation. Recent Dev Microb Technol..

[46] Hawley AK, Nobu MK, Wright JJ, Durno WE, Morgan-Lang C, Sage B (2017). Diverse *Marinimicrobia* bacteria may mediate coupled biogeochemical cycles along eco-thermodynamic gradients. Nat Commun.

[47] Jaekel U, Musat N, Adam B, Kuypers M, Grundmann O, Musat F (2013). Anaerobic degradation of propane and butane by sulfate-reducing bacteria enriched from marine hydrocarbon cold seeps. ISME J.

[48] Thapa HR, Naik MT, Okada S, Takada K, Moln NI, Xu Y (2016). A squalene synthase-like enzyme initiates production of tetraterpenoid hydrocarbons in *Botryococcus braunii*. Nat Commun.

[49] Zadjelovic V, Chhun A, Quareshy M, Silvano E, Hernandez-Fernaud JR, Aguilo-Ferretjans MM (2020). Beyond oil degradation: enzymatic potential of *Alcanivorax* to degrade natural and synthetic polyesters. Environ Microbiol.

[50] Zakem EJ, Al-Haj A, Church MJ, van Dijken GL, Dutkiewicz S, Foster SQ (2018). Ecological control of nitrite in the upper ocean. Nat Commun.

[51] Pachiadaki MG, Sintes E, Bergauer K, Brown JM, Record NR, Swan BK (2017). Major role of nitrite-oxidizing bacteria in dark ocean carbon fixation. Science.

[52] Hazen TC, Prince RC, Mahmoudi N (2016). Marine oil biodegradation. Environ Sci Technol.

[53] Cruaud P, Vigneron A, Fradette M-S, Charrette S, Rodriguez M, Dorea CC (2017). Open the Sterivex™ casing: an easy and effective way to improve DNA extraction yields. Limnol Oceanogr Methods.

[54] Bolger AM, Lohse M, Usadel B (2014). Trimmomatic: a flexible trimmer for Illumina sequence data. Bioinformatics.

[55] Nawrocki EP, Eddy SR. Infernal 1.1: 100-fold faster RNA homology searches. Bioinformatics. 2013;29:2933–5. 10.1093/bioinformatics/btt509 PMC3810854 24008419

[56] Pruesse E, Quast C, Knittel K, Fuchs BM, Ludwig W, Peplies J (2007). SILVA: a comprehensive online resource for quality checked and aligned ribosomal RNA sequence data compatible with ARB. Nucleic Acids Res.

[57] Bankevich A, Nurk S, Antipov D, Gurevich AA, Dvorkin M, Kulikov AS (2012). SPAdes: a new genome assembly algorithm and its applications to single-cell sequencing. J Comput Biol.

[58] Bushnell B. BBMap: a fast, accurate, splice-aware aligner. Ernest Orlando Lawrence Berkeley National Laboratory, Berkeley, CA (US); 2014.

[59] Markowitz VM, Mavromatis K, Ivanova NN, Chen I-MA, Chu K, Kyrpides NC. IMG ER: a system for microbial genome annotation expert review and curation. Bioinformatics. 2009;25:2271–8. 10.1093/bioinformatics/btp393 19561336

[60] Li D, Liu C-M, Luo R, Sadakane K, Lam T-W (2015). MEGAHIT: an ultra-fast single-node solution for large and complex metagenomics assembly via succinct de Bruijn graph. Bioinformatics.

[61] Kang DD, Froula J, Egan R, Wang Z (2015). MetaBAT, an efficient tool for accurately reconstructing single genomes from complex microbial communities. PeerJ.

[62] Parks DH, Imelfort M, Skennerton CT, Hugenholtz P, Tyson GW (2015). CheckM: assessing the quality of microbial genomes recovered from isolates, single cells, and metagenomes. Genome Res.

[63] Chaumeil P-A, Mussig AJ, Hugenholtz P, Parks DH (2020). GTDB-Tk: a toolkit to classify genomes with the Genome Taxonomy Database. Bioinformatics.

[64] Sievers F, Higgins DG (2018). Clustal Omega for making accurate alignments of many protein sequences. Protein Sci.

[65] Minh BQ, Schmidt HA, Chernomor O, Schrempf D, Woodhams MD, von Haeseler A (2020). IQ-TREE 2: new models and efficient methods for phylogenetic inference in the genomic era. Mol Biol Evol.

