# Supplementary material for: Genomic insights into cryptic cycles of microbial hydrocarbon production and degradation in contiguous freshwater and marine microbiomes
Source: Microbiome. 2023 May 12;11:104. doi: 10.1186/s40168-023-01537-7 (PMC10176705; doi:10.1186/s40168-023-01537-7)
Supplement: Supplementary file 2 — Additional file 1: Supplementary Fig. S1. Detail of the genomic MAGs analysed in this study. Completeness and contamination were determined using ChekM. Maximum coverage was calculated using the average coverage determined using BWA-MEM of all contigs found in the MAGs. Green dots indicate the presence in the MAGs of hydrocarbon production genes while orange and blue dots refer to hydrocarbon degradation genes. Pink and purple dots indicate genes involved in nitrogen and sulfur transformations respectively. Detail of the genes (KO) identified in the MAGs is available in Supplementary Dataset 1. [file 40168_2023_1537_MOESM1_ESM.docx]

**Supplementary Material**

**Genomic insights into cryptic cycles of microbial hydrocarbon production and degradation in contiguous freshwater and marine microbiomes**

Adrien Vigneron^1,2,3,4*^, Perrine Cruaud^3,5^, Connie Lovejoy^1,3,4,6^ and Warwick F. Vincent^1,2,3,4^

**Supplementary Figure 1:** Detail of the genomic MAGs analysed in this study. Completeness and contamination were determined using ChekM. Maximum coverage was calculated using the average coverage determined using BWA-MEM of all contigs found in the MAGs. Green dots indicate the presence in the MAGs of hydrocarbon production genes while orange and blue dots refer to hydrocarbon degradation genes. Pink and purple dots indicate genes involved in nitrogen and sulfur transformations respectively. Detail of the genes (KO) identified in the MAGs is available in Supplementary Dataset 1.

**Supplementary Dataset:** Detail of the genes (KO) identified in the MAGs involved in hydrocarbon production and degradation.

**Supplementary Figure 1**
